# Morbidity after reversal of Hartmann operation: retrospective analysis of 56 patients

**Published:** 2015

**Authors:** EC Zarnescu (Vasiliu), NO Zarnescu, R Costea, L Rahau, S Neagu

**Affiliations:** * Second Department of Surgery, University Emergency Hospital Bucharest, Romania; ** Department of Surgery, “Carol Davila” University of Medicine and Pharmacy, Bucharest, Romania; *** “Carol Davila” University of Medicine and Pharmacy, Bucharest, Romania

**Keywords:** colostomy closure, Hartmann’s reversal, morbidity, anastomotic fistula, anastomotic leakage

## Abstract

**Background:** Despite patient selection, postoperative morbidity after reversal of Hartmann’s procedure remains significant.

**Aim:** The objective of this study was to investigate risk factors associated with morbidity after conversion of Hartmann’s operation.

**Patients and methods:** We retrospectively analyzed data of 56 patients who underwent reversal procedures between January 2004 and May 2015 in a single center. We evaluated the following variables: demographic characteristics, medical comorbidities, etiology for Hartmann operation, preoperative lab values, intraoperative surgical details and short-term outcomes (hospital stay, medical and surgical complications, mortality).

**Results:** There were 37 men (66.1%) and the mean age was 57 years. The most frequent indications for Hartmann’s procedure were colorectal cancer in 25 patients (44.6%) and complicated diverticulitis in 10 patients (17.9%). The mean time to the reversal procedure was 9 months. Morbidity rate was 16.1% (9 patients) with an anastomotic leakage rate of 3.6% (2 patients) and mortality rate was 3.6% (2 patients). The most common medical complication was diarrhea (4 patients, 7.2%). Bivariate analysis demonstrated that the only factor significantly associated with postoperative complications was presence of multiple comorbidities.

**Conclusions:** Multiple medical comorbidities is the only predictive factor for postoperative complications after Hartmann’s reversal and therefore patient selection for this type of surgery is critical.

## Introduction

The Hartmann’s procedure was originally indicated for complicated cancer of the lower sigmoid and upper rectum, but today it is used for a variety of indications, such as perforated diverticulitis, traumatic perforations, volvulus, inflammatory colitis, and postoperative anastomotic leaks. It is most often used in emergency colorectal surgery in patients in whom an immediate anastomosis is not feasible and in those believed to be at a very high risk for anastomotic leakage. Boyden was the first who described the use of Hartmann’s reversal procedure in the surgical management of acute diverticulitis [**1**].

The importance of Hartmann’s reversal results in increasing the quality of life (QoL). Vermeulen et al. assessed long-term QoL in a cohort of 76 patients after Hartmann’s procedure or primary anastomosis [**2**]. They found that QoL in patients who underwent Hartmann’s procedure was lower compared to primary anastomosis. After Hartmann’s reversal, this difference disappeared, but reversal was performed in only 61% of the patients.

After recovery from the initial Hartmann operation, colostomy reversal with restoration of bowel continuity was a procedure of a high degree of complexity. This second stage requires a major abdominal surgery and is associated, according to different authors, with a complication rate ranging from 0.2% to 60.0% [**3**,**4**]. In this setting, the anastomotic leakage can reach 12.0% [**2**]. Such high level of complications leads to a high mortality, ranging 4.0-10.0% [**3**-**5**]. 

A high frequency of complications and mortality is caused by different reasons, such as massive postoperative adhesive process in the abdomen which can arise after Hartmann’s procedure, the low locating of the rectal stump and its involving in adhesions or distortion of normal anatomic planes. A big distance between anastomosed parts requires, in some cases, additional mobilization of a proximal colon. In some situations, there is a necessity of colon re-resection, for example, for diverticulosis or recurrence of the colon cancer.

Unfortunately, restoration of intestinal continuity by reversal of the Hartmann’s procedure remains a technically challenging operation that can be performed in only one third of these patients [**5**,**6**]. Reasons for not reversing the stoma include age, ASA score, high-risk status, patient refusal, and a fear of postoperative complications [**3**,**5**-**7**]. The purpose of this paper is to review the postoperative clinical outcome of the conversion of Hartmann procedure.

## Material and methods

We reviewed the medical charts of 56 patients who were admitted in our department between 2004 and 2015 for reversal of Hartmann’s procedure. All data was retrospectively abstracted from de-identified patient records. Preoperative data for analysis included patient’s age, gender, medical comorbidities, standard blood values, indication of the Hartmann’s operation and the period between Hartmann’s procedure and reversal. Intraoperative data contained type of surgical technique. Postoperative outcomes included length of hospital stay, number of ICU (intensive care unit) admissions, postoperative complications (medical and surgical) and mortality. 

**Statistical analysis**

All values were presented as mean and standard deviation or proportions. Bivariate comparisons of postoperative complications (surgical and/ or medical complication) were assessed by using the Mann-Whitney U test for continuous variables and Fisher-exact test for categorical variables. A two-sided p value of <0.05 was considered to be significant. Statistical analyses were performed by using SPSS software package, version 15 (SPSS Inc, Chicago, IL).

## Results

The study population consisted of 56 patients who underwent Hartmann’s reversal between January 2004 and May 2015. There were 37 men (66.1%) and 19 women (33.9%) with a mean age of 57+15.6 years. The most frequent indications for Hartmann’s procedure were colorectal cancer in 25 patients (44.6%) and complicated diverticulitis in 10 patients (17.9%). Colo-rectal cancer location was rectal in 6 patients (24%), sigmoid in 13 patients (52%) and more proximal colon in 6 patients (24%). The mean time until reversal was 9+7 months.

**Table 1:** Indications for Hartmann’s procedure in 56 patients with restoration of intestinal continuity

Hartmann’s indications	Number of patients (%)	Interval (months) Hartmann – reversal (mean+SD)
Cancer	25 (44.6%)	11 ± 5.5
Rectal	6 (24%)	
Sigmoid	13 (52%)	
Other colon location	6 (24%)	
Complicated diverticulitis	10 (17.9%)	5.3 ± 3.7
Anastomotic leakage	4 (7.1%)	6.7 ± 2.5
Other indications	17 (30,4%)	8.9 ± 7

Medical comorbidities were present in 23 patients (41.1%); Seven patients (14.9%) had multiple comorbidities. The most frequent comorbidities were arterial hypertension in 14 patients (25%) and diabetes mellitus in 6 patients (10.7%). Preoperative lab values were hemoglobin 13.7+1.4g/ dl, total proteins 7.4+0.5g/ dl, glucose 101+23 mg/ dL, BUN 35+15 mg/ dL, creatinine 0.9+0.3 mg/ dL. All the reversal procedures were performed by using the open approach. The anastomosis was made hand sewn in 44 cases (78.6%), while the remaining 12 cases were stapled. The anastomosis was performed: end-to-end in 32 cases (57.1%), end-to-side in 15 patients (26.8%), side-to-end in 5 patients (8.9%) and side-to-side in 2 patients (3.6%).

The mean length of hospital stay was 11 + 2.4 days. There were 9 complications (16.1%) (**Table 2**). Two patients (3.6%) died. Out of four patients with diarrhea, only one patient was diagnosed with Clostridium difficile infection. Anastomotic leakage was diagnosed in 2 patients (3.6%), after 7 and 8 days; the treatment was conservative for both of them. One patient had both diarrhea syndrome and anastomotic leakage. The other patient with anastomotic fistula further evolved with septic syndrome and later died. One patient was admitted in ICU department due to myocardial infarction and died 24 hours after admission.

**Table 2:** Outcomes after Hartmann’s reversal

Outcomes	Number
Hospital duration of stay (days)	11 ± 2.4
Number of ICU admission	1 (1.8%)
Complications*	9 (16.1%)
Anastomotic leakage	2 (3.6%)
Wound infection	1 (1.8%)
Diarrhea syndrome	4 (7.2%)
Cardiovascular complication	2 (3.6%)
Hemorrhagic gastritis	1 (1.8%)
Death	2 (3.6%)
*one patient had two complications	

The bivariate analysis of postoperative complications is described in **Table 3**. Due to a low number of surgical complications (3 patients) and mortality (2 patients), such an analysis could not be performed.

**Table 3:** Bivariate analysis of postoperative complications of 56 patients with Hartmann’s reversal

Variables	No complications (N = 47)	Postoperative complications (N=9)	p-value
Male gender (N=37)	29 (61.7%)	8 (88.9%)	0.113
Age (years)	56.8+14.7	58.5+20	0.510
Medical comorbidities (N=23)	18 (38.3%)	5 (55.6%)	0.274
Multiple comorbidities (N=7)	4 (8.5%)	3 (33.3%)	0.039*
Hartmann - conversion interval (months)	8.5+6.3	11.8+10	0.277
Hartmann’s indication			0.900
Cancer (N=25)	21 (44.7%)	4 (44.4%)	
Diverticulitis (N=10)	8 (17%)	2 (22.2%)	
Anastomotic leakage (N=4)	3 (6.4%)	1 (11.1%)	
Other indications (17)	15 (31.9%)	2 (22.2%)	
Cancer location – sigmoid (N=13)	11 (52.4%)	2 (50%)	0.996
Manual surgical technique (N=44)	37 (78.7)	7 (77.8%)	0.949
Type of anastomosis			0.474
End to end (N=32)	27 (60%)	5 (55.6%)	
End to side (N=15)	11 (24.4%)	4 (44.4%)	
Hemoglobin (g/ dL)	13.7±1.4	14.1±1.7	0.369
Total proteins (g/ dL)	7.4±0.5	7.3±0.3	0.755
*p<0.05			

## Discussions

Although primary resection (for colorectal cancer or colonic diverticulosis) with anastomosis has been increasingly performed in recent years, many surgeons may still prefer multistage procedures in the presence of complications, especially in emergency cases. Restoration of gastrointestinal continuity depends on several factors such as patient’s request, local factors, general condition and comorbidities or potential length of survival. Reversal rate of Hartmann’s procedure is reported in most studies between 4% and 85% and are higher for benign pathology [**7**-**11**].

The most common indication for Hartmann’s procedure in our study was colorectal cancer, comparable with other studies [**11**,**12**]. In our study of 56 patients, reversal for cancer represented 44.6% compared with 17.9% for diverticulitis that reflected a higher proportion of cancer as a primary indication for Hartmann procedure compared with diverticulitis found in western population. However, Desay at al. reported in their study a reversal for cancer of only 13% compared to 70% for diverticulitis [**13**]. There is no consensus regarding the timing of reversal of Hartmann’s procedure. Many studies reported a median time to the reversal procedure of 9 months that is similar with our findings. Delayed reversal has been advocated in several studies, the reasons included less dense adhesions and more time to optimize the clinical and nutritional status of the patient [**5**,**6**,**11**]. In our patients, time until reversal was shorter for diverticulitis compared with cancer (6 months vs. 12 months). One explanation for delayed reversal in cancer could be the adjuvant chemo-radiotherapy treatment in these patients. In our study, there was no significant association between the timing of reversal and postoperative complications.

The median length of stay was 11 days, similar to that reported in most other series [**13**-**15**]. The postoperative morbidity of 16.1% in our series was comparable with the morbidity rates quoted in literature. The anastomotic leakage rate was 3.6% that was comparable to previous results reported in other studies [**5**,**7**,**10**,**16**]. The advanced age (over 80) has been found as a risk factor for increased surgical morbidity and mortality in some studies [**17**,**18**]. Our study found no correlation between age and postoperative complications. There was no relation between type of anastomosis and morbidity. We found no statistical relevance between morbidity and mortality and etiology as reported in other studies [**11**,**16**,**19**].

**Table 4:** Morbidity after reversal of Hartmann’s operation

Study	No of patients	Anastomotic leakage rate (%)	Morbidity (%)
Banerjee et al. (2005) [**7**]	63	4 (6.34%)	26 (41%)
Fleming et al. (2009) [**20**]	76	1 (1.31%)	18 (25%)
Schmelzer et al. (2007) [**19**]	113	1 (0.88%)	28 (24.7%)
Tokode et al. (2011) [**16**]	51	4 (7.8%)	19 (37.3%)
Tan et al. (2012) [**12**]	46	0 (0.0%)	10 (20.4%)
Our study	56	2 (3.6%)	9 (16.1%)

The most frequent complication was diarrheic syndrome in 4 patients (7.2%). Only one patient was diagnosed with Clostridium difficile infection and developed anastomotic leakage on the seventh postoperative day, which was treated conservatively. Due to the small number of patients, we could not demonstrate any correlations between infection with Clostridium difficile and anastomotic fistula. Anastomotic leakage was an uncommon complication in our study (3.6%). One patient with anastomotic fistula died on the twelfth day postoperatively due to a septic condition and multiple cardio-vascular comorbidities. In our analysis, the only statistically significant factor associated with morbidity was the presence of multiple comorbidities.

In conclusion, restoration of intestinal continuity after Hartmann’s procedure appears to be a safe operation with low morbidity and mortality. Multiple medical comorbidities appear to be the only predictive factor for postoperative complications and therefore patient selection for this operation is critical.
